# Soluble prebiotic fiber and glucono-δ-lactone blends integrate compatible effects to modulate microbiota composition and metabolism *ex vivo*

**DOI:** 10.3389/fmicb.2026.1923298

**Published:** 2026-10-08

**Authors:** Damien Truffin, Caroline Perreau, Liam Jenkins Sánchez, Jasmine Heyse, Aurélien Baudot, Lam Dai Vu, Emma De Beul, Pieter Van den Abbeele

**Affiliations:** 1 Roquette Frères, Lestrem, France; 2 Cryptobiotix SA, Ghent, Belgium

**Keywords:** gut microbiome, host-microbiome interactions, metabolomics, short-chain fatty acids (SCFA), soluble prebiotic fiber

## Abstract

**Introduction:**

Prebiotics modulate the gut microbiome toward health-associated states. Because distinct candidate prebiotic compounds selectively stimulate microbial taxa and metabolic pathways, combining complementary ingredients may broaden or enhance beneficial outcomes. To investigate this concept, we evaluated the effects of microbiome-modulating blends of glucono-δ-lactone (GDL) and the resistant dextrin NUTRIOSE^®^ (NUTR).

**Methods:**

The validated *ex vivo* SIFR^®^ technology pipeline (systemic intestinal fermentation research) was used to assess how GDL, NUTR and their blends impacted the composition and metabolic activity of the gut microbiota of a small cohort of healthy adults (*n* = 6), while effects on gut barrier integrity (transepithelial electrical resistance, TEER) were evaluated via an *in vitro* co-culture of epithelial/immune (Caco-2/THP-1) cells.

**Results:**

GDL and NUTR induced distinct yet compatible shifts in microbial composition and metabolite output. GDL was rapidly fermented and specifically promoted *Bifidobacterium adolescentis*, while boosting butyrate-related taxa (*Faecalibacterium prausnitzii*, *Anaerobutyricum hallii*) and health-associated amino acid derivatives (e.g., indole-3-propionic acid). In contrast, NUTR gradually and strongly promoted propionate through stimulating *Bacteroides* and *Parabacteroides*, as well as specific Lachnospiraceae taxa. GDL/NUTR blends displayed ratio-dependent intermediate effects of the individual ingredients. Moreover, GDL/NUTR blends enhanced gut barrier integrity under LPS-induced inflammatory stress, with increases in TEER up to +24.5%.

**Discussion:**

Overall, GDL and NUTR blends compatibly integrate divergent effects, supporting their combined use for enhancing gut health through microbiome modulation.

## Graphic abstract

**Figure:**
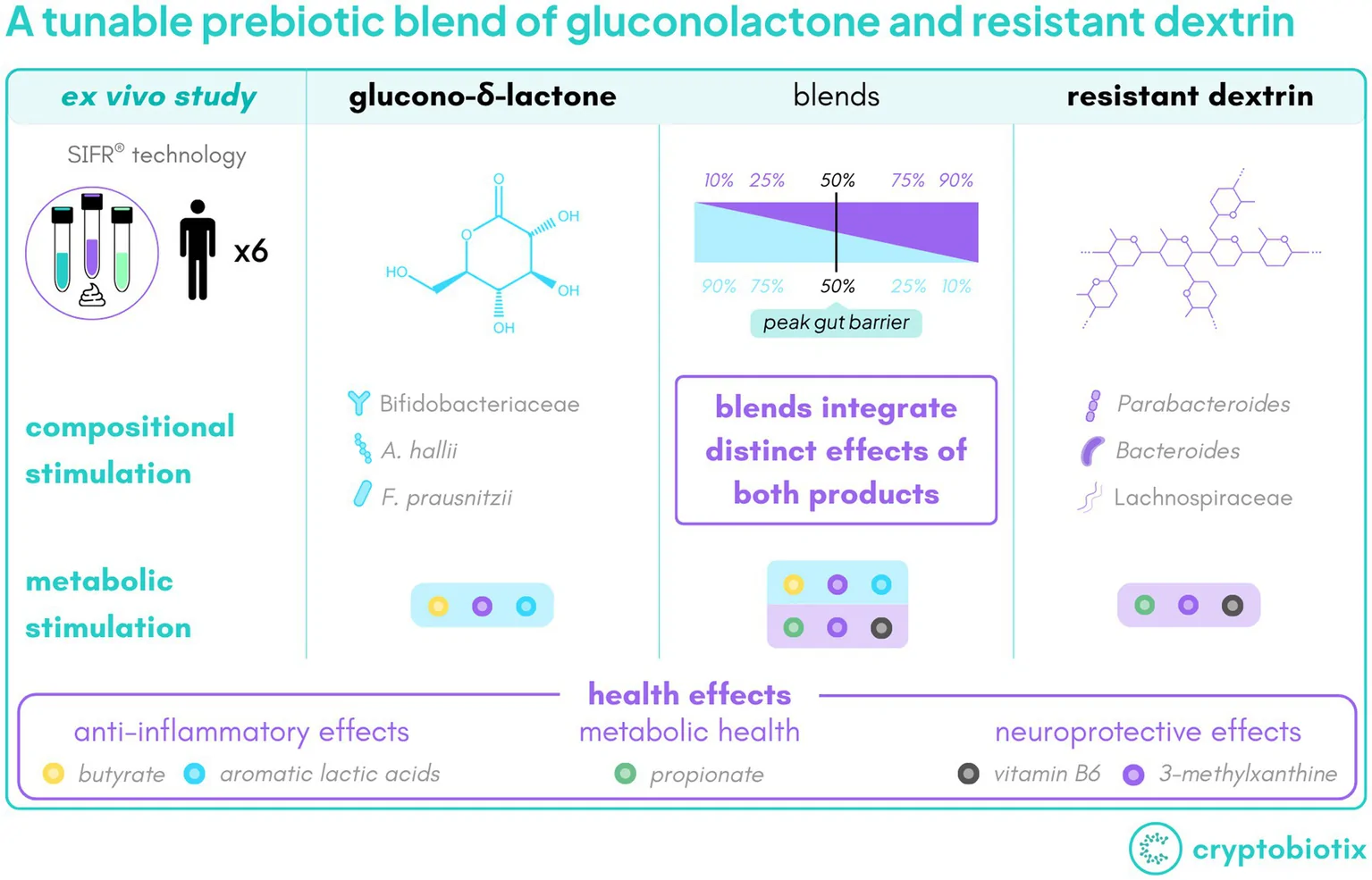

## 1 Introduction

The gut microbiota is an integral component of the human digestive system, and as such, its metabolic state profoundly influences host well-being. Consequently, several approaches have been developed to modulate the gut microbiome toward favorable conditions, among which prebiotics are particularly prominent. Prebiotics are defined as substrates that are selectively utilized by host microorganisms conferring a health benefit (Gibson et al., 2017). For instance, they can selectively stimulate beneficial intestinal microbes or induce favorable metabolic equilibria, thereby promoting host health. Most prebiotics are saccharic in nature, such as the fructan inulin (Davani-Davari et al., 2019). These non-digestible carbohydrates are often fermented by the gut microbiome resulting in the production of short-chain fatty acids (SCFA), mainly acetate, propionate and butyrate (Louis and Flint, 2017), which each in turn interact with the host gut lining (Morrison and Preston, 2016; Rivière et al., 2016). However, prebiotic effects can be highly compound-dependent.

Glucono-δ-lactone (GDL) is a cyclic ester of gluconic acid and is a well-known food additive (Kuwano et al., 2018). Beyond its technological applications, emerging evidence suggests that gluconic acid and related compounds can modulate gut microbial composition and activity (Cañete-Rodríguez et al., 2016; Michiels et al., 2023; Negishi et al., 2025), hinting at potential prebiotic activity. Human, animal and *in vitro* studies reported stimulation of the prominent butyrate producer *Faecalibacterium prausnitzii* or enhanced butyrate production (Michiels et al., 2023; Negishi et al., 2025). Notably, butyrate is the preferred energy source for colonocytes (Louis and Flint, 2017), which may explain why gluconic acid administration has been associated with improved hallmarks for gut health in porcine studies, including heightened anti-inflammatory signaling (Michiels et al., 2023). Conversely, prebiotics with glycan-like structures tend to exert different effects. NUTRIOSE^®^ (NUTR) is an established prebiotic polysaccharide composed of resistant dextrin, forming a large yet soluble polymer. *In vivo*, NUTR administration has been proven to enhance satiety in adult humans (Guerin-Deremaux et al., 2011), while *ex vivo* colonic simulations showed that NUTR fermentations elevate acetate and propionate levels, via the promotion of *Parabacteroides distasonis* (Van den Abbeele et al., 2023). GDL and NUTR each act on the microbiota in beneficial yet strongly divergent manners. For this reason, the combined use of both products is an intriguing prospect. By simultaneously stimulating two sets of health-related pathways, complementary activities may be achieved. Additionally, blends of these two microbiome-modulating products could exhibit mixed characteristics, allowing for the selection of an intermediate formulation with fine-tuned properties.

The *ex vivo* SIFR^®^ technology pipeline (systemic intestinal fermentation research) is well-suited to address this research goal as it enables studying the human gut microbiota and host interactions under physiologically relevant conditions. By adopting a high-throughput workflow, interindividual variability can be captured across test subjects. Most importantly, it provides predictive insights into clinical outcomes, including microbiome modulation and gut barrier function (Van den Abbeele et al., 2023; Van den Abbeele et al., 2024).

The present study aimed to discern the individual and combined effects of GDL and NUTR on the human adult gut microbiota, as well as their potential benefits on epithelial integrity. Using the *ex vivo* SIFR^®^ technology pipeline, we observed differential yet compatible effects of the products on the microbiota and demonstrated positive effects on gut barrier integrity, with GDL/NUTR blends delivering a balanced impact. This highlights the potential of combining a fast-fermenting ingredient with a more complex fiber for integrated effects.

## 2 Materials and methods

### 2.1 Test products

The compounds of interest were glucono-δ-lactone (GDL, Roquette, Lestrem, France; 99% pure, batch SE564) and NUTRIOSE^®^ FM HF (NUTR, Roquette, Lestrem, France; 4% moisture, on dry basis: 97% fiber and 1% mono-/disaccharides, average degree of polymerization: 13.8 ± 1.0, dispersity ≈ 1.8, batch E322N). A total of five combinations of GDL and NUTR were blended into mixed products at ratios of 90/10, 75/25, 50/50, 25/75 and 10/90 by weight to cover the mixture range at regular intervals, resulting in products 90G:10N, 75G:25N, 50G:50N, 25G:75N and 10G:90N, respectively. The unmixed and blended products were compared with a parallel unsupplemented control (no-substrate control, NSC). All test compounds and blends were evaluated at a dose equivalent to a human intake of 5 g/day.

### 2.2 Fecal microbiota sourcing

To simulate biorelevant gut microbiota *ex vivo* using the SIFR^®^ technology, fresh fecal samples were obtained from six healthy adults [mean age: 45.0 ± 13.5 years; test subject 1 (male, 38), 2 (female, 36), 3 (male, 62), 4 (female, 40), 5 (female, 65) and 6 (female, 29)]. These subjects had not used antibiotics within 3 months before donation, were non-smoking, reported no use of probiotics, exhibited a BMI under 30, consumed under 3 units of alcohol per day and had no history of gastrointestinal disorders (cancer, ulcers, inflammatory bowel disease). The study received approval from the Ethics Committee of the University Hospital Ghent (reference number BC-09977; approval date: 13 April 2021). All test subjects provided written informed consent for anonymous use of their fecal samples and scientific publication of anonymized study results.

### 2.3 *Ex vivo* colonic fermentation (SIFR^®^)

*Ex vivo* experiments were performed as previously reported (Van den Abbeele et al., 2023). In brief, fecal samples were individually subjected to an anaerobic batch incubation. Each bioreactor (type 1 borosilicate glass) contained a total liquid volume of 5 mL, comprising (i) a nutritional medium (M0017, available from Cryptobiotix, Ghent, Belgium), which contained approximately 1 g/L carbohydrates, 1.9 g/L organic nitrogen sources and 0.9 g/L mucin to support a broad diversity of gut microorganisms (corresponding to an estimated C: N ratio of around 5), while providing an *in vivo*-relevant buffering capacity, (ii) a fecal slurry (prepared from a freshly collected fecal sample from a single test subject, on average introducing 1.4 × 10^9^ bacterial cells/mL) and (iii) the appropriate test product(s). The initial pH of the inoculated medium was 6.77 ± 0.02. The bioreactors were rendered anaerobic using a bioreactor management device (Cryptobiotix, Ghent, Belgium) by replacing the reactor atmosphere with oxygen-free nitrogen gas. Following anaerobization, the reactors were sealed, removed from the bioreactor management device and incubated under continuous agitation (140 rpm) at 37 °C for up to 48 h (MaxQ 6000, Thermo Scientific, Merelbeke, Belgium). Independent 5 mL bioreactors were prepared for each sampling time point (0 h, 6 h, 24 h and 48 h), with individual reactors harvested at their designated time point.

Eight study arms were tested for each subject (*n* = 6): NSC, GDL, five different GDL/NUTR blends at various ratios by weight (90G:10N, 75G:25N, 50G:50N, 25G:75N and 10G:90N) and NUTR. At 0 h (NSC only) and after 6 h, 24 h and 48 h of incubation, headspace gas pressure was measured, and liquid samples were collected to investigate key fermentative parameters. Microbial taxonomic composition and untargeted metabolomics were analyzed at 0 h for NSC and at 48 h for all study arms. Host-microbiome interaction analysis was performed using samples collected after 48 h of fermentation (Figure 1). Treatment effects for each test product were evaluated by comparing with NSC at the same time point (6 h, 24 h or 48 h).

**Figure 1:**
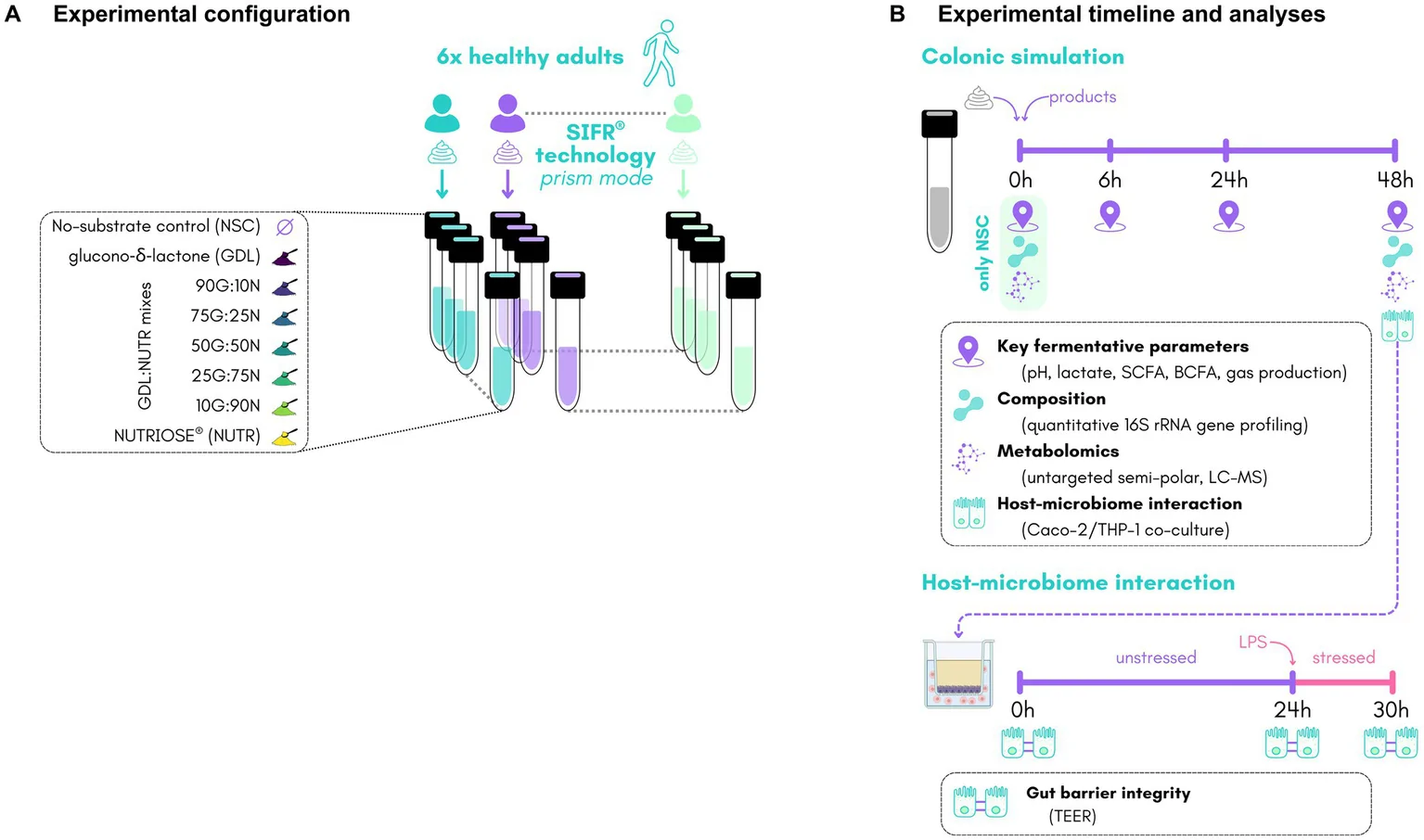
Experimental design for investigating the effects of glucono-δ-lactone (GDL) and NUTRIOSE^®^ (NUTR) blends on the microbiota of 6 healthy adult donors using the *ex vivo* SIFR^®^ technology. **(A)** Colonic fermentation setup. **(B)** Timepoints for sampling and analytics. BCFA, branched-chain fatty acids, LC-MS, liquid chromatography-mass spectrometry; LPS: lipopolysaccharide, NSC, no-substrate control; SCFA, short-chain fatty acids, SIFR^®^, systemic intestinal fermentation research; TEER, transepithelial electrical resistance.

The high technical reproducibility of the SIFR^®^ technology (Van den Abbeele et al., 2023) was confirmed by running NSC study arms in technical triplicate for each test subject and measuring key fermentative parameters after 48 h. The coefficients of variation for pH, gas and SCFA (acetate, propionate, butyrate) measurements were 0.8% for technical replicates and 8.4% for biological replicates, reflecting high reproducibility and expected interindividual differences.

### 2.4 Analysis of key fermentative parameters

Sample pH was measured using an electrode (Hannah Instruments Edge HI2002, Temse, Belgium). Lactate was quantified using an enzymatic method (Enzytec™, R-Biopharm, Darmstadt, Germany). Acetate, propionate, butyrate, valerate, isobutyrate, isocaproate and isovalerate were extracted using diethyl ether and quantified using gas chromatography with flame ionization detection, as previously described (Van den Abbeele et al., 2023). Briefly, 0.5 mL samples were diluted in distilled water (1:3), acidified with 0.5 mL of 48% sulfuric acid, and supplemented with an excess of sodium chloride, 0.2 mL of internal standard (2-methylhexanoic acid) and 2 mL of diethyl ether. After homogenization and phase separation, the diethyl ether layer was collected and analyzed using a Trace 1300 chromatograph (Thermo Fisher Scientific, Merelbeke, Belgium) equipped with a Stabilwax-DA capillary GC column, a flame ionization detector, and a split injector using nitrogen gas as the carrier and makeup gas. The injection volume was 1 μL and the temperature ranged from 110 °C to 240 °C. The temperatures of the injector and detector were 240 and 250 °C, respectively. Total SCFA was defined as the sum of acetate, propionate, butyrate, valerate and BCFA (branched-chain fatty acids), with BCFA as the sum of isobutyrate, isovalerate and isocaproate. As SCFA formation is often accompanied by gas production, the gas-to-SCFA ratio was calculated as an indicator of fermentation tolerability by dividing gas production (mBar) by total SCFA levels (mM).

### 2.5 Taxonomic characterization of microbiota composition

Quantitative 16S rRNA gene profiling was used to investigate changes in microbiota composition at 0 h (NSC only) and 48 h (all study arms). Total DNA was extracted from each microbiota sample using the SPINeasy DNA Kit for Soil (MP Biomedicals, Eschwege, Germany), according to manufacturer’s instructions. The 16S rRNA gene V3-V4 hypervariable regions were amplified using primers 341F (5’-CCT ACG GGN GGC WGC AG-3′) and 785Rmod (5’-GAC TAC HVG GGT ATC TAA KCC-3′) appended with Illumina adapter sequences. The products were subjected to a second PCR using sample-specific barcoded primers (Nextera XT index kit; Illumina, San Diego, CA, United States), followed by multiplexing, clustering and sequencing on an Illumina MiSeq platform with v3 chemistry (2×300 bp) (Illumina, San Diego, CA, USA). Reads were demultiplexed with bcl2fastq v2.20, adapter and primer sequences were removed, and paired-end reads were merged using BBMerge v34.48. Sequences were subsequently processed in mothur v1.35.1 (Schloss et al., 2009). Reads containing ambiguous bases, homopolymers stretches of more than 8 bases or an average Phred quality score below 33 were removed. Sequences were aligned against the 16S mothur-Silva SEED r119 reference alignment, preclustered allowing up to one mismatch per 100 bases, and chimeras were eliminated using the UCHIME algorithm (Edgar et al., 2011). OTUs were clustered at 97% sequence identity using the average-neighbor method, and singleton OTUs were excluded. Representative OTU sequences were subsequently compared against the Genome Taxonomy Database (GTDB) (Parks et al., 2020), release 220, for taxonomic assignment. No correction for 16S rRNA gene copy numbers was applied. The final outputs were analyzed at phylum, family, genus or OTU level as appropriate. To convert proportional data to representative absolute cell counts (cells/mL), flow cytometry analysis was performed. Samples were diluted in anaerobic phosphate-buffered saline (PBS), stained with SYTO 16 (Life Technologies Europe NV, Merelbeke, Belgium) at a final concentration of 1 μM, and counted via a BD FACS Verse flow cytometer (BD, Erembodegem, Belgium). The data were analyzed using FlowJo, version 10.5.0. Quantitative insights into microbial composition were obtained by combining relative abundances (%) obtained via 16S rRNA gene profiling with total bacterial counts (cells/mL) obtained via flow cytometry, resulting in estimated cell densities (cells/mL) for different taxonomic levels.

### 2.6 Untargeted analysis of microbial metabolite production

Metabolomics was performed through liquid chromatography-mass spectrometry (LC-MS), using a Vanquish UPLC device coupled to a Q Exactive™ HF Hybrid Quadrupole-Orbitrap (both from Thermo Fisher Scientific), using an electrospray ionization source. Analysis was performed in negative and positive ionization mode. The analysis followed a slightly modified version of the protocol described by Doneanu et al. (2011). Peak areas were extracted using Compound Discoverer 3.3 (Thermo Scientific) in combination with a manual extraction of compounds included in an in-house library using Skyline 21.1 (MacCoss Lab Software) (Adams et al., 2020), and reported without normalization. To ensure correctly annotated metabolites, the data analysis focused on metabolites annotated based on a combination of retention time matching (using in-house authentic standards) and accurate mass (with an accepted deviation of 3 ppm), and MS/MS spectral information where available. Accurate mass and MS/MS spectra were measured with high resolution Orbitrap ESI-MS in mzCloud (Thermo Fisher Scientific), MassBank of North America (UC Davis) and the European MassBank (Helmholtz Centre for Environmental Research Leipzig). Further, the analysis was limited to compounds that increased in at least 4 out of the 6 test donors in at least one treatment between 0 h and 48 h, suggesting they are actively produced by gut microbes or present in the test products. Technical reproducibility was assessed by including a QC sample (pooled sample of all samples) every six injections. These QC samples clustered together in the exploratory analysis of confidently annotated metabolites, confirming the high technical reproducibility of the method, with an average coefficient of variation of 8.2%.

### 2.7 Host-microbiome interaction assay

A co-culture model was used to investigate how the effects of the test products on the gut microbiota (as assessed via the SIFR^®^ technology) may impact the host colonic epithelium, specifically the gut barrier integrity (Van den Abbeele et al., 2024). The cell model consisted of human epithelial cells (human adenocarcinoma Caco-2 cell line, ATCC^®^ HTB-37™) and immune cells (human acute monocytic leukemia THP1 cell line, differentiated to activated macrophages by a phorbol 12-myristate 13-acetate (PMA) treatment, ATCC^®^ TIB-202™), both derived from the American Type Culture Collection (ATCC, Manassas, VA, USA).

Briefly, upon differentiation over 14 and 2 days for Caco-2 and THP-1 cells, respectively, the immune cells were covered with an epithelial layer in a permeable well insert to recreate a model gut wall. Colonic samples derived from the 48 h colonic incubation were centrifuged and filter-sterilized before administration (20 v/v%) on the apical side of the co-culture model. Additionally, control conditions (without colonic fermentation supernatant) were included for incubation. These were 2 blanks with medium only, with or without lipopolysaccharide (LPS, Invitrogen, Carlsbad, CA, USA) added after 24 h, and 2 positive controls combining LPS with either dexamethasone (D) or hydrocortisone (HC), both representing strong anti-inflammatory compounds.

The host-microbiome interaction assay consisted of two phases: (i) 24 h treatment with colonic samples (or controls) to evaluate gut barrier integrity under unstressed conditions, and (ii) an additional 6 h incubation after administering 500 ng/mL lipopolysaccharide (LPS) on the basal side to evaluate behavior under stressed conditions. Gut barrier integrity was assessed by measuring transepithelial electrical resistance (TEER) at 0 h, 24 h (before LPS addition) and 30 h (after LPS treatment). TEER values at 24 h and 30 h were normalized to the baseline (0 h) measurement of the corresponding well to account for well-to-well variability in initial barrier resistance.

### 2.8 Statistical analysis

Data analysis was performed using R (version 4.4.3; www.r-project.org) (R Core Team, 2026). Violin plots, dot plots, line charts, bar charts and heatmaps were assembled using the ggplot2 package (v4.0.0) (Wickham, 2016). For exploratory evaluation of baseline microbial composition, principal component analysis (PCA) was performed with the FactoMineR package (Lê et al., 2008). Values of all accounted genera were standardized (mean = 0, standard deviation = 1) and the two principal components with largest eigenvalues were plotted. Statistical analysis was conducted using a linear mixed-effects model, which accounted for both fixed effects (treatment) and random effects (variation between donor samples) using the glmmTMB package v1.1.11 (Brooks et al., 2017). Differences between treatments were assessed via *post hoc* pairwise comparison using estimated marginal means (emmeans package v1.11.2). The statistical significance of potential treatment effects was corrected via Benjamini-Hochberg (Benjamini and Hochberg, 1995) correction for false discovery rate (FDR = 0.05, stats package v4.6). Statistical differences versus the NSC at each matched timepoint were visualized via * (0.01 < *p*_adjusted_ < 0.05), ** (0.001 < *p*_adjusted_ < 0.01) or *** (*p*_adjusted_ < 0.001) in the figures, while all pairwise comparisons are provided in Supplementary Table S1.

For the statistical evaluation of the effects on microbial composition and metabolite production, the linear mixed-effects model was first applied to test overall effect of treatments on taxa/metabolites, and only the taxa/metabolites that displayed significant overall treatment effects were further considered for *post hoc* pairwise comparison. For taxonomic abundances below the limit of quantification (LOQ), numerical values were set to the LOQ before log_2_-transformation. The LOQ was calculated by dividing one read by the total amount of reads in each sample, followed by multiplication with the bacterial cell count detected via flow cytometry. This provided a LOQ for each sample individually. Then, the highest LOQ was used as overall LOQ of the entire dataset. The community modulation score (Tintoré et al., 2024) (CMS) was calculated at genus level, using the 69 most abundant genera (covering on average 98.5% of the microbial communities). The CMS measures the balance between the number of genera that are increased and those that are suppressed by a treatment compared to the NSC. A positive CMS therefore indicates that a treatment stimulates a broader range of genera than it suppresses. Although CMS does not directly quantify richness or evenness and is thus distinct from conventional α-diversity indices, such a positive balance represents a diversity-supporting community response that, if sustained during prolonged treatment, would be expected to promote greater microbial diversity over time. Pairwise correlation analysis was performed between compositional data (log_2_-transformed abundance at family level) and concentrations of the three main SCFA (acetate, propionate, butyrate) using Spearman’s rank correlation coefficient, with Benjamini-Hochberg correction of *p*-values as described above.

## 3 Results

### 3.1 Taxonomic analysis of fecal microbiota from six donors showcased diverse profiles

The baseline composition of the fecal microbiota of six healthy human adults was characterized to assess interindividual variability within the study cohort (Figure 2). Principal component analysis (PCA) of genus-level abundances revealed substantial heterogeneity between donor microbiotas. Donors 1, 3 and 6 clustered together due to sharing higher *Prevotella* levels, while donors 2, 4 and 5 showed *Bacteroides*/*Phocaeicola*-dominated profiles. Donors 2 and 4 were additionally characterized by elevated *Agathobacter* abundance, whereas *Faecalibacterium* was particularly abundant in donors 1, 3, 5 and 6. Donor 2 showed the highest *Blautia_A* abundance, while donor 3 displayed high levels of *Megamonas* and *Holdemanella*. Together, these observations indicate that the donor cohort encompassed distinct microbiota profiles, consistent with patterns previously reported in larger human cohorts (Arumugam et al., 2011; Costea et al., 2018) and enabling the evaluation of treatment effects across diverse gut microbial backgrounds.

**Figure 2:**
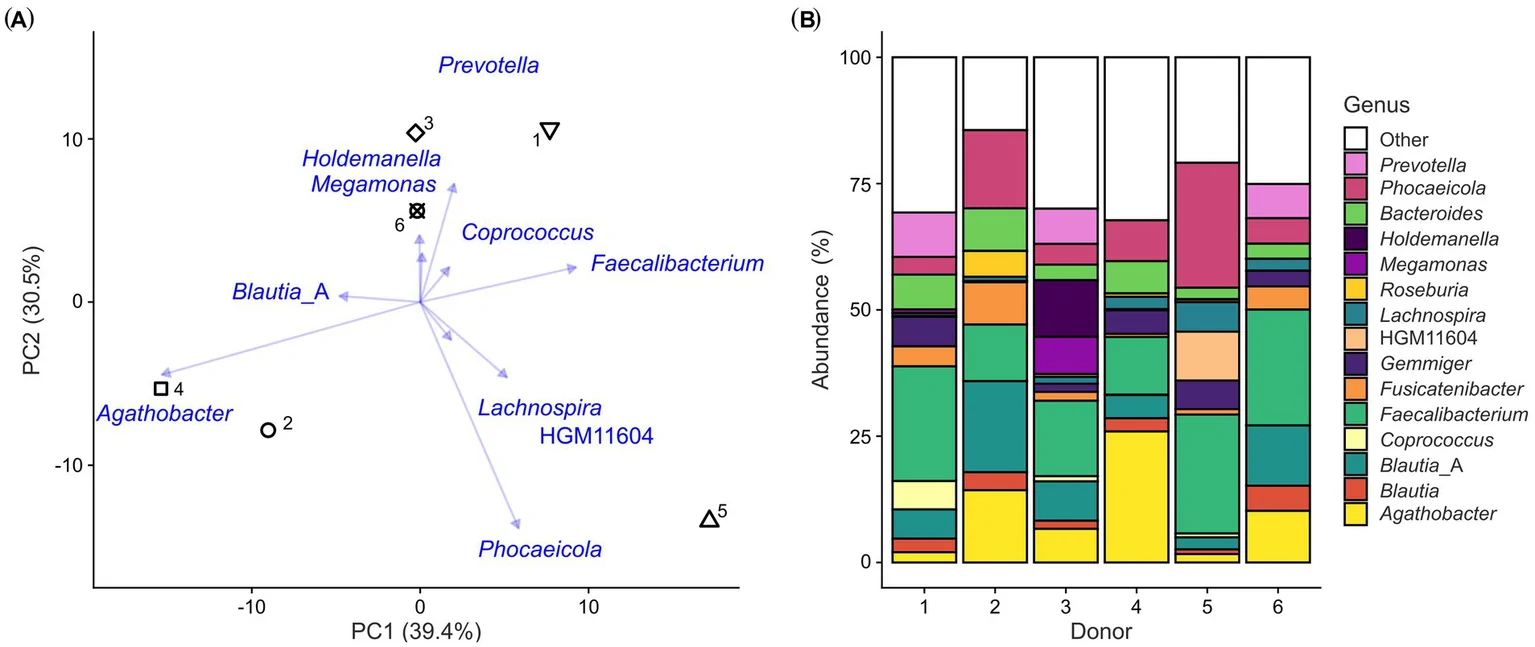
Fecal microbiota from six adult donors exhibited high interindividual variation in microbial composition. **(A)** Principal component analysis (PCA) based on standardized relative abundance at the genus level. **(B)** Abundance (%) of the 15 most abundant genera in the fecal microbiota of the six donors.

### 3.2 GDL and NUTR stimulated different gut microbial taxa in an integrated manner

Colonic fermentation simulations using the *ex vivo* SIFR^®^ technology enabled the study of GDL, NUTR and their blends as modulators of the gut microbiota composition. Observations at the phylum level showcased distinct impacts of GDL and NUTR, while blends exhibited intermediate effects (Figure 3A). GDL supplementation selectively enriched Actinomycetota, while NUTR promoted broad outgrowth with a strong preference for Bacillota_A and Bacteroidota members. Blends of GDL and NUTR integrated features of both individual ingredients with microbiota profiles progressively shifting toward a GDL- or NUTR-like pattern depending on their relative proportions, suggesting additive prebiotic effects.

**Figure 3:**
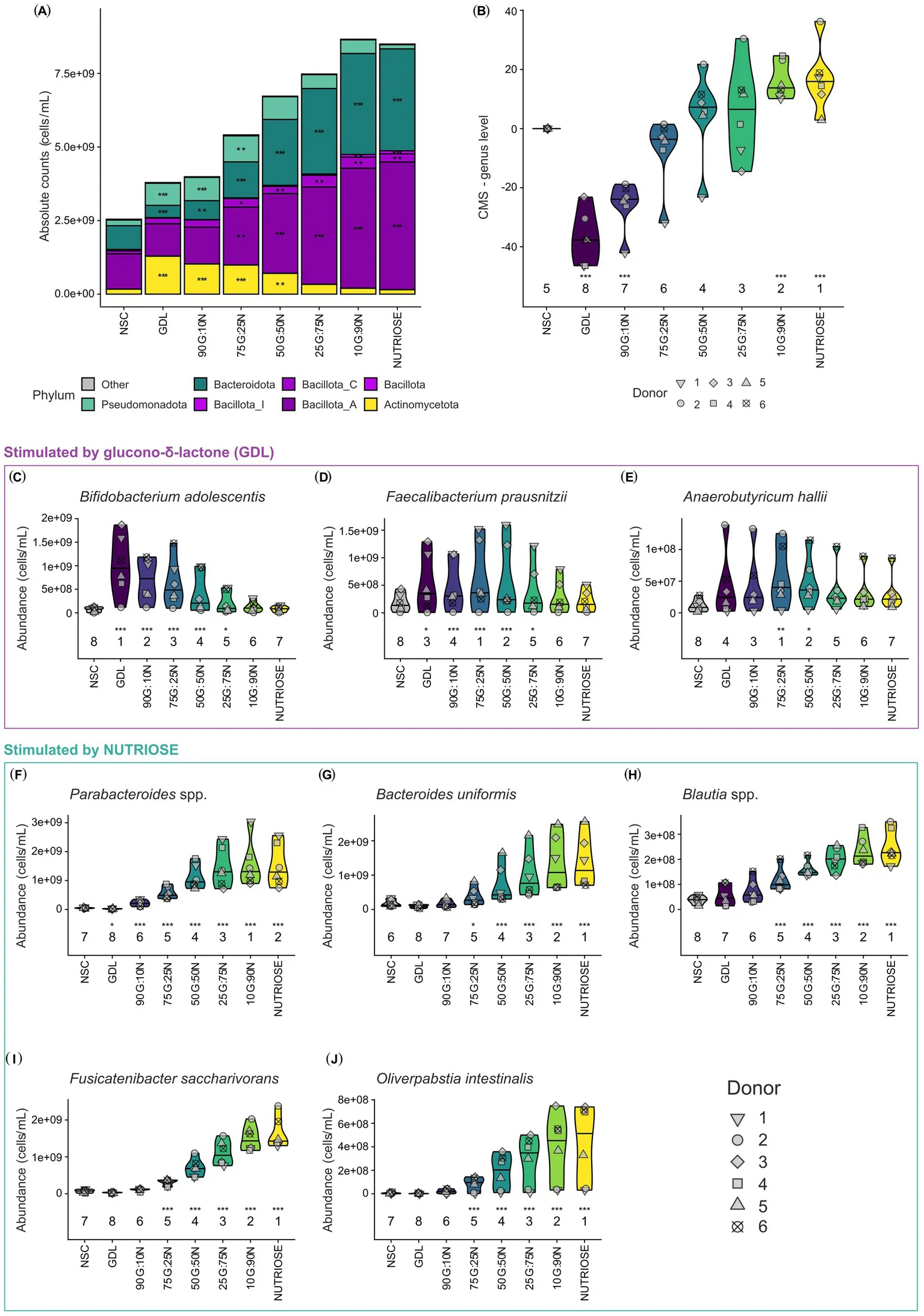
Glucono-δ-lactone (GDL) and NUTRIOSE^®^ (NUTR) exerted compatible effects on microbiota composition after 48 h of *ex vivo* colonic fermentation. **(A)** Average absolute abundance (cells/mL) of bacterial phyla after simulated colonic fermentation per treatment, compared to a no-substrate control (NSC). **(B)** Community modulation score (CMS) (Tintoré et al., 2024) per treatment, showing donors as replicates. **(C–J)** Absolute abundance (cell/mL) of individual selected OTUs and genera per treatment: **(C)** *Bifidobacterium adolescentis*, **(D)** *Faecalibacterium prausnitzii*, **(E)** *Anaerobutyricum hallii*, **(F)** *Parabacteroides* spp., **(G)** *Bacteroides uniformis*, **(H)** *Blautia* spp., **(I)** *Fusicatenibacter saccharivorans*, and **(J)** *Oliverpabstia intestinalis*. Statistical differences between NSC and individual treatments at the same timepoint are indicated with * (0.01 < *p*_adjusted_ < 0.05), ** (0.001 < *p*_adjusted_ < 0.01) or *** (*p*_adjusted_ < 0.001). The ranks of the average values per treatment are indicated as well, with 1 corresponding to the highest average value and 8 corresponding to the lowest average value.

At the genus level, GDL and NUTR distinctly modulated the gut microbiota, as measured by the CMS, which reflects the balance between stimulated and suppressed genera relative to the NSC (Figure 3B) (Tintoré et al., 2024). Notably, GDL administration lowered the CMS compared to NSC due to its narrow stimulation of specific bacterial genera (Supplementary Figure S1). In contrast, CMS increased progressively with increasing NUTR content in the blends, significantly exceeding the NSC from 90% NUTR. Pairwise comparisons between the treatments further demonstrated a stepwise increase in CMS, with significant differences between consecutive blend ratios, except for 50G:50N versus 25G:75N and 10G:90N versus NUTR (Supplementary Table S1). This reflects the broader prebiotic activity of NUTR, simultaneously promoting a more diverse range of bacterial genera.

Detailed investigation at family, genus and OTU level (Figures 3C–J; Supplementary Figures S2, S3; Supplementary Table S1) revealed taxa that were selectively modulated by the various treatments. GDL tended to specifically promote the outgrowth of *Bifidobacterium adolescentis* (Actinomycetota). Additionally, GDL stimulated a narrow spectrum of butyrate-producing bacteria from the Bacillota_A phylum, concretely *Faecalibacterium prausnitzii* (significant) and *Anaerobutyricum hallii* (not significant). Beyond these taxa, GDL exerted relatively limited growth-promoting effects, with only a few additional taxa, such as *Veillonella dispar* and *Escherichia*, showing increased abundances compared to the NSC. Conversely, NUTR blends exerted broader growth-promoting effects. Generally, the Bacteroidota phylum was stimulated, including taxa within Tannerellaceae (*Parabacteroides distasonis* but also *P. merdae* and *P. goldsteinii*) and Bacteroidaceae (mainly *Bacteroides uniformis*), many of which prolific propionate producers. Likewise, numerous taxa of the Bacillota_A phylum were promoted, especially those of the Lachnospiraceae family. These tended to be glycan fermenters and SCFA producers, such as *Blautia* spp., *Fusicatenibacter saccharivorans, Oliverpabstia intestinalis, Gallintestinimicrobium propionicum, Anthropogastromicrobium aceti* and *Roseburia* species.

Interestingly, the effects of GDL and NUTR were combined in a ratio-dependent manner in blends (Figures 3C–J; Supplementary Figures S2, S3). Taxa preferentially stimulated by either ingredient generally exhibited gradual, dose-dependent responses across the blend series, resulting in microbiota profiles that integrated characteristic features of both GDL and NUTR. Notable exceptions were *F. prausnitzii* and *A. hallii*. The blends 75G:25N and 50G:50N showed the strongest stimulation of these two OTUs, with effects not significantly different from GDL alone (Supplementary Table S1). This suggests that inclusion of NUTR did not diminish the GDL-mediated stimulation of these butyrate-producing taxa and may even have slightly enhanced it.

### 3.3 Fermentative parameters were compatibly affected by GDL and NUTR

The pronounced effects of GDL and NUTR on gut microbiota composition were accompanied by associated changes in fermentative profiles (Figure 4, Supplementary Figures S4, S5; Supplementary Table S1). All products and blends resulted in elevated gas and SCFA levels and reduced pH and BCFA formation after 48 h of fermentation, compared to the NSC. However, GDL and NUTR exhibited marked differences in fermentation speed and SCFA profile. GDL/NUTR blends showed intermediate effects, with fermentative parameters exhibiting shifts according to product ratios, indicating that the fermentation characteristics of both ingredients were preserved within the blends.

**Figure 4:**
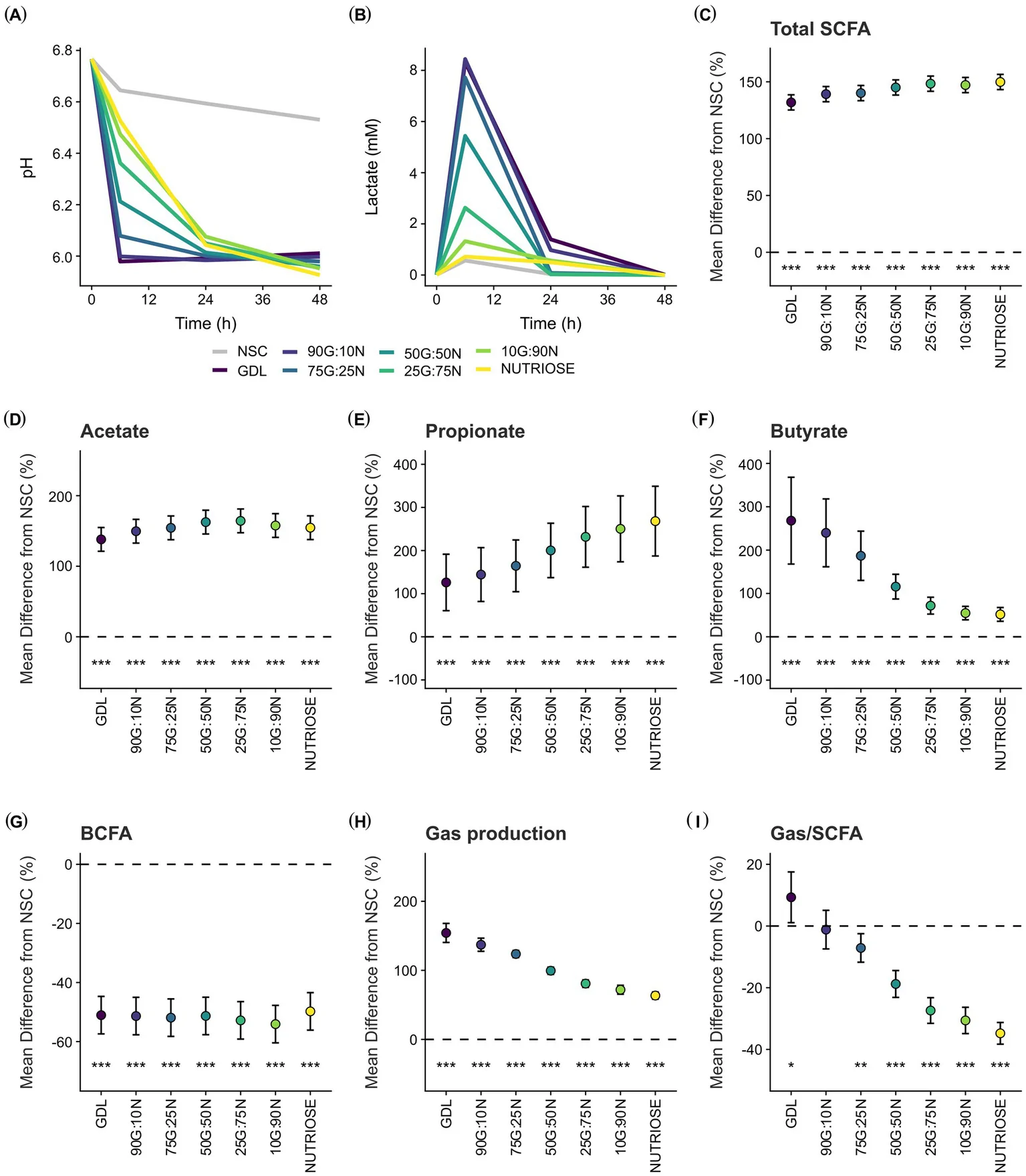
Fermentative parameters differentially shifted upon glucono-δ-lactone (GDL) or NUTRIOSE^®^ (NUTR) administration, with integrated effects for their blends. **(A,B)** Impact of GDL, NUTR and their blends on **(A)** pH and **(B)** lactate at different timepoints throughout their simulated colonic fermentation by the gut microbiota of healthy adults. Values are presented as the average among the six donors. **(C–I)** Impact on **(C)** total short-chain fatty acids (SFCA), **(D)** acetate, **(E)** propionate, **(F)** butyrate, **(G)** branched-chain fatty acids (BCFA), **(H)** gas production, and **(I)** gas-to-SCFA ratio after 48 h of simulated colonic fermentation by the gut microbiota of healthy adults (*n* = 6). Values are expressed as the percentage change relative to the no-substrate control (NSC; dashed line) as estimated by the regression outputs from the linear mixed models. Error bars represent 95% confidence intervals. Statistical differences between NSC and treatments at the same timepoint are indicated with * (0.01 < *p*_adjusted_ < 0.05), ** (0.001 < *p*_adjusted_ < 0.01) or *** (*p*_adjusted_ < 0.001). Time-course data for total SCFA, acetate, propionate, butyrate, gas production and BCFA are shown in Supplementary Figure S5.

Treatment with GDL and GDL-rich blends led to a rapid decrease in pH with concomitantly high lactate levels after 6 h (Figures 4A,B). This was accompanied by rapid gas formation, particularly during the first hours of fermentation, as well as strong acetate production by GDL-rich blends at 6 h (Supplementary Figures S4, S5; Supplementary Table S1). By 48 h, lactate had largely been consumed (Figure 4B), likely through microbial cross-feeding into SCFA. Among these SCFA, GDL-rich treatments preferentially stimulated butyrate production, with GDL, 90G:10N and 75G:25N yielding significantly higher butyrate levels than all other treatments, both at 24 h and 48 h (Figure 4F; Supplementary Figure S5D; Supplementary Table S1). Interestingly, butyrate concentrations after 48 h showed a strong and significant positive correlation with Bifidobacteriaceae abundance (Supplementary Figure S6C).

In contrast, NUTR was more slowly fermented. Effects on pH and lactate levels were more gradual and restrained for NUTR, as was gas formation (Figures 4A,B; Supplementary Figures S4, S5E; Supplementary Table S1). Although NUTR induced the lowest SCFA production at 6 h, SCFA levels increased steadily over time and significantly exceeded those of GDL-rich treatments by 48 h (Figure 4C; Supplementary Figure S5A; Supplementary Table S1). Accordingly, fermentations with NUTR showed a lower gas-to-SCFA ratio, an effect already significant compared to NSC from 25% NUTR onwards (Figure 4I). This implies a potentially superior *in vivo* tolerability profile for blends containing this fiber. Unlike GDL, NUTR specifically promoted propionate production, with treatments of at least 50% NUTR yielding significantly more propionate at 48 h than GDL-rich blends (Figure 4E; Supplementary Figure S5C; Supplementary Table S1). Propionate levels positively associated with the stimulation of Bacteroidaceae and Tannerellaceae (Supplementary Figures S6A,B). By 48 h, acetate concentrations were of similar magnitude across all treatments (Figure 4D), although levels tended to be slightly higher with 50G:50N and 25G:75N (only significant vs. GDL, Supplementary Table S1).

### 3.4 Metabolomics analysis revealed health-related metabolites promoted by GDL and NUTR

Untargeted metabolomics was performed to identify further metabolites whose production was triggered by GDL and NUTR, beyond traditional fermentation parameters. Of the 136 confidently annotated compounds, 64 metabolites increased in at least 4 of the 6 donors during the 48 h fermentation and were retained for analysis. A total of 34 compounds was significantly stimulated by at least one treatment relative to NSC (Supplementary Figure S7; Supplementary Table S1). GDL and NUTR again displayed distinct responses, while blends integrated these effects in an additive and dose-dependent manner. GDL specifically stimulated indole-3-lactic acid and indole-3-propionic acid, which are both derived from tryptophan catabolism (Figures 5A,B). Additionally, hydroxyphenyllactic acid, the lactic acid derivative of tyrosine, was also boosted by GDL, as well as the structurally related 3-(3-hydroxyphenyl)propionic acid (Figures 5C,D). Conversely, NUTR was associated with increases of several aromatics, such as *trans*-3-hydroxycinnamic acid, salicylic acid, benzoic acid and atenolol (Figures 5G–J). The tryptophan derivative *N*-acetyltryptophan and the vitamin B6 vitamer pyridoxamine were also stimulated by NUTR (Figures 5K,L). Some metabolites were enhanced by both, e.g., 4-guanidinobutyric acid and 3-methylxanthine (Figures 5E,F).

**Figure 5:**
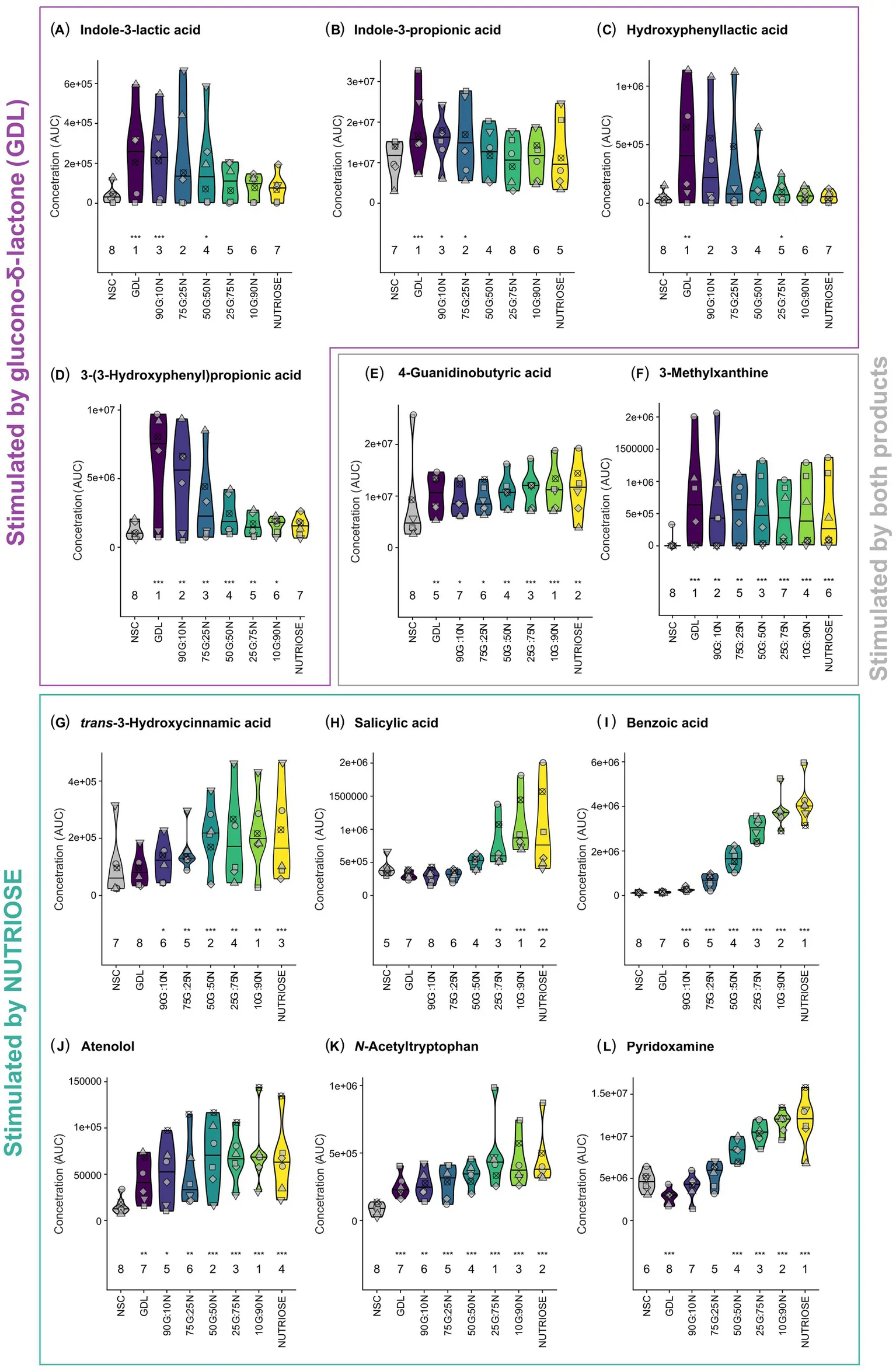
Health-related metabolites were specifically stimulated by glucono-δ-lactone (GDL), NUTRIOSE^®^ (NUTR) and their blends. Concentration of health-related metabolites in response to treatments compared to a no-substrate control (NSC) after 48 h of simulated colonic fermentation by the gut microbiota of healthy adults (*n* = 6), expressed in Area Under Curve (AUC). Selected metabolites were preferentially stimulated **(A–D)** by GDL, **(G–L)** by NUTR or **(E,F)** by both products. Statistical differences compared to the NSC at the same timepoint are indicated with * (0.01 < *p*_adjusted_ < 0.05), ** (0.001 < *p*_adjusted_ < 0.01) or *** (*p*_adjusted_ < 0.001). The ranks of the average values per treatment are indicated as well, with 1 corresponding to the highest average concentration and 8 corresponding to the lowest average concentration.

Beyond the metabolites annotated with high confidence, interesting responses were also observed among metabolites with lower annotation support. In particular, compounds tentatively identified as 5-indolol and 2-oxobutyric acid were strongly promoted by NUTR and are known to exert *in vivo* health effects. These findings may warrant further investigation, as annotation confidence was low in the current analysis.

### 3.5 GDL and NUTR blends enhanced gut barrier integrity upon LPS-induced stress

A Caco-2/THP-1 co-culture model was used to validate whether the microbiome modulation by GDL and NUTR translated to improved gut health. For this purpose, the co-culture model was incubated for 24 h with the supernatants of 48 h SIFR® colonic fermentations. After this 24-h incubation, transepithelial electrical resistance (TEER) was measured as a proxy for gut barrier integrity. Subsequently, LPS was added to the basal side to mimic an inflammatory state, and the co-cultures were incubated for a further 6 h before once more measuring TEER. This enabled uncovering whether supernatants from GDL/NUTR fermentations aid in protecting gut barrier integrity upon inflammation.

As expected, after a total incubation of 30 h, the LPS-treated negative control showed a lower TEER compared to the unexposed blank, reflecting a compromised epithelial barrier (Figure 6B). Conversely, anti-inflammatory compounds dexamethasone (D) or hydrocortisone (HC) mitigated the LPS-induced reduction in TEER, restoring barrier integrity to levels comparable to those observed in the blank control, thereby validating the challenge model.

**Figure 6:**
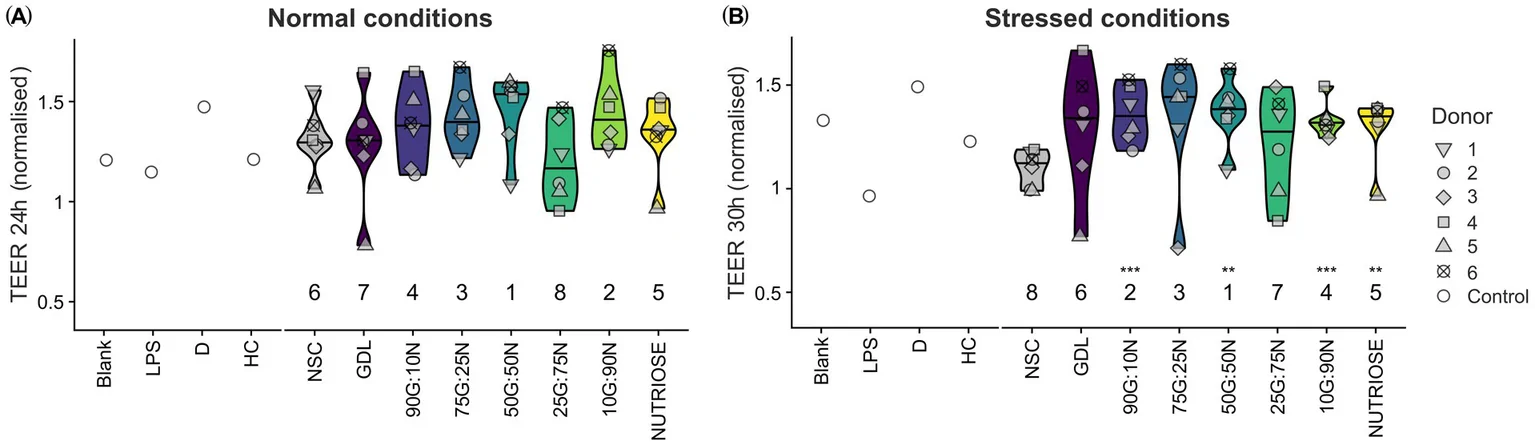
Glucono-δ-lactone (GDL) and NUTRIOSE^®^ (NUTR) blends promote gut barrier integrity under stressed conditions. **(A)** Transepithelial electrical resistance (TEER) in a Caco-2/THP-1 co-culture after 24 h of incubation with supernatants derived after 48 h of simulated colonic fermentation by the gut microbiota of healthy adults (*n* = 6). **(B)** TEER values after an additional 6 h incubation under inflammatory conditions induced via basal lipopolysaccharide application. LPS: lipopolysaccharide negative control, D: dexamethasone with LPS positive control, HC: hydrocortisone with LPS positive control. Statistical differences between NSC and individual treatments at each matched timepoint are indicated with * (0.01 < *p*_adjusted_ < 0.05), ** (0.001 < *p*_adjusted_ < 0.01) or *** (*p*_adjusted_ < 0.001). The ranks of the average values per treatment are indicated as well, with 1 corresponding to the highest TEER value and 8 corresponding to the lowest average TEER value.

In the unstressed model before basal LPS challenge, co-culture models incubated with GDL/NUTR fermentation supernatants did not show significant differences in TEER relative to the NSC (Figure 6A). However, under LPS-stressed conditions, all test products tended to maintain a higher TEER compared to NSC (Figure 6B). In particular, 90G:10N, 50G:50N, 10G:90N and NUTR significantly increased TEER by 23.7% (*p*_adjusted_ < 0.0001), 24.5% (*p*_adjusted_ = 0.0014), 21.1% (*p*_adjusted_ < 0.0001) and 17.3% (*p*_adjusted_ = 0.0017) compared to NSC, respectively. This suggests some GDL/NUTR blends may exert a particularly strong protective effect on gut barrier integrity in inflammation states, although the differences between treatments were not statistically significant (Supplementary Table S1).

## 4 Discussion

The aim of this study was to investigate the impact of GDL, NUTR and different combinations thereof on the human adult gut microbiota, via the *ex vivo* SIFR^®^ technology pipeline. All products exhibited strong effects across each of the analyzed aspects of the colonic simulations, with GDL and NUTR each shifting results in specific patterns, while blended treatments integrated these characteristic effects into intermediate outcomes. In general, both GDL and NUTR strongly affected the human microbiome *ex vivo*. Changes in fermentative parameters indicated a metabolically active microbiota and implied a shift away from proteolytic (reduced BCFA levels) and toward saccharolytic fermentation (Davila et al., 2013), suggesting good fermentability. Likewise, microbial composition analysis showed that GDL and especially NUTR stimulated specific gut microbes, in a strongly product-dependent manner.

GDL was rapidly fermented, with strong effects on pH and pronounced gas, acetate and lactate formation already after 6 h. Its effects on microbial composition were relatively targeted, as reflected by a decrease in CMS. The strongest response was observed for *B. adolescentis*, suggesting that this OTU was a primary contributor to GDL fermentation and likely responsible for the high initial acetate and lactate production. Besides its bifidogenic effect, GDL also promoted the butyrate producers *F. prausnitzii* (Moens et al., 2016) and *A. hallii* (Bunesova et al., 2018; Belenguer et al., 2006) while preferentially stimulating butyrate formation after 48 h of fermentation. Together, these observations point toward a cross-feeding mechanism, where acetate and lactate produced during bifidobacterial fermentation are subsequently converted into butyrate by secondary fermenters (Moens et al., 2016; Belenguer et al., 2006). Promotion of *F. prausnitzii* has previously been reported following gluconic acid supplementation in a pig study (Michiels et al., 2023), supporting the reproducibility of this response across experimental systems.

NUTR fermentation exhibited different characteristics, reflecting its more complex polymeric structure. Compared to GDL, NUTR was fermented more gradually, with moderate effects on lactate and pH, and only low gas production. This may indicate improved tolerability, given rapid gas formation is associated with bloating and discomfort *in vivo*. In contrast to the focused activity of GDL on certain microbiota taxa, NUTR exerted broader growth-promoting effects, resulting in a higher CMS through stimulation of a more diverse range of microbial genera. NUTR promoted several taxa previously linked to resistant dextrin fermentation, such as *Parabacteroides* spp., *Bacteroides uniformis*, *Blautia* spp. and *Fusicatenibacter saccharivorans* (Zhang et al., 2021; Reichardt et al., 2018; Nishimoto et al., 2022). Broad stimulatory effects were observed toward Bacteroidota, which are notable propionate producers (Louis and Flint, 2017; Shin et al., 2024). Indeed, Bacteroidota (Tannerellaceae and Bacteroidaceae) abundance correlated with propionate levels, likely contributing to the preferential stimulation of propionate formation by NUTR and NUTR-rich blends. Specifically, the promotion of *Parabacteroides distasonis* by NUTR has been previously reported both *in vivo* and in another SIFR^®^ study (Van den Abbeele et al., 2023; Thirion et al., 2022). Interestingly, members of the Bacteroidota phylum have been associated with anti-inflammatory and favorable immune responses in various disease contexts (Ramakrishna et al., 2019; Shen et al., 2012; Vernay et al., 2020; Johnson et al., 2015; Routy et al., 2018). In particular, *Parabacteroides* species have attracted interest as next-generation probiotics given their remarkable anti-inflammatory effects and anti-obesity potential (Wang et al., 2019; Cuffaro et al., 2020).

Various other NUTR-stimulated taxa belonged to the Bacillota_A phylum, most prominently in the Lachnospiraceae. Many are important SCFA-producers, such as *Roseburia* (Louis and Flint, 2017), *Oliverpabstia intestinalis*, *Fusicatenibacter saccharivorans* (Takada et al., 2013), and *Gallintestinimicrobium propionicum*. Further, several *Blautia* strains have been studied for their probiotic potential against metabolic diseases (Xu et al., 2023; Hosomi et al., 2022), and inhibitory effects on lipid accumulation, weight gain, and blood glucose elevation (Guo et al., 2024), hinting toward health-promoting effects of NUTR administration.

It was a notable and repeated finding that blends of GDL and NUTR simultaneously showcased activities associated with both individual ingredients, rather than displaying competitive behavior. This is promising, as divergent effects can be integrated into more balanced products with desired characteristics. For example, increasing the proportion of NUTR in blends appeared to slow down fermentation and attenuate gas formation, suggesting mixed products with adjusted fermentation speed and gas formation characteristics can be obtained by modulating the GDL/NUTR ratio. Effects on microbial composition can also be integrated, as NUTR-promoted bacteria increased already from 25% NUTR onward, whereas taxa associated with GDL generally required higher GDL concentrations, with pronounced impacts from 50G:50N. These findings suggest that blends of 75G:25N or 50G:50N may provide a balanced profile and integrate compatible effects on microbial communities influenced by both GDL and NUTR. Interestingly, some taxa responded particularly well to blended formulations. For example, partial replacement of GDL by NUTR tended to further increase the abundance of the butyrate producers *F. prausnitzii* and *A. hallii* beyond the effect of GDL alone. Additionally, blends of GDL and NUTR in different proportions resulted in varying butyrate/propionate ratios. This implies formulations can be adjusted to promote either of these SCFA preferentially and do so in a balanced manner.

A balanced SCFA profile is particularly relevant as each brings its own clinical health benefits (Blaak et al., 2020; Xiong et al., 2022; Mansuy-Aubert and Ravussin, 2023; Facchin et al., 2024; Silva et al., 2020). Notably, all treatments boosted acetate, which is a minor energy source for epithelial cells and is rapidly incorporated into tissues after reaching the portal vein. Butyrate, the main SCFA stimulated by GDL, is the preferred fuel for colonocytes and plays a key role in maintaining gut barrier integrity by strengthening tight junctions and stimulating mucin production. This SCFA may also be protective against colon cancer and colitis (Rivière et al., 2016). Interestingly, increased butyrate production following gluconic acid supplementation was previously reported in newly weaned piglets (Michiels et al., 2023). Conversely, NUTR administration tended to specifically elevate propionate, which *in vivo* is taken up by the liver and contributes to metabolic health by promoting satiety, lowering cholesterol and improving insulin sensitivity (Rivière et al., 2016). Given the divergent health-promoting pathways, simultaneous activation of these effects may be desirable.

Furthermore, GDL and NUTR stimulated distinct metabolite profiles beyond SCFA, as revealed by untargeted metabolomics. GDL-rich treatments modulated tryptophan and tyrosine metabolism, steering it toward the production of aromatic lactic acids such as indole-3-lactic acid and hydroxyphenyllactic acid, as well as their related propionic acids indole-3-propionic acid and 3-(3-hydroxyphenyl)propionic acid. Interestingly, these metabolites have previously been associated with various health benefits, including antioxidant, anti-inflammatory, neuroprotective and cardiometabolic effects (Negatu et al., 2020; Chyan et al., 1999; Hwang et al., 2009; Serger et al., 2022; Kim et al., 2023; de Mello et al., 2017; Kasperek et al., 2023; Myers et al., 2026; Van Duynhoven et al., 2013; Larrosa et al., 2009; Jiang et al., 2022; Najmanová et al., 2016), as well as roles in muscle and bone health (Du et al., 2021; Kong et al., 2025; Liu et al., 2026; Chen et al., 2019).

Likewise, NUTR-rich treatments promoted a distinct metabolite profile, including stimulation of health-associated compounds. For example, *N*-acetyltryptophan has been linked to immunoprotective effects (Sirianni et al., 2015), while benzoic acid is a known redox regulator (Mao et al., 2019). The stimulation of pyridoxamine by NUTR was of particular interest given the well-established role of vitamin B6 in host metabolism and gut homeostasis (Ali et al., 2022; Kennedy, 2016; Hossain et al., 2022; Ligaarden and Farup, 2011).

Some metabolites were promoted by both GDL and NUTR. In particular, 3-methylxanthine and 4-guanidinobutyric acid were consistently elevated across all treatments. Methylxanthines are known to exhibit antioxidant, neuroprotective and cardioprotective activity (Monteiro et al., 2016; Oñatibia-Astibia et al., 2017), while 4-guanidinobutyric acid protects against gastric lesions (Hwang and Jeong, 2012) and is a precursor for γ-aminobutyric acid (Foster and Kemp, 2006).

Together, these findings highlight distinct metabolic signatures associated with GDL and NUTR fermentation, suggesting that both ingredients may exert beneficial effects through modulation of the gut metabolome, although physiological relevance of the observed metabolite shifts remains to be evaluated.

Given the stimulation of various health-related mechanisms by GDL and NUTR, it was of interest to evaluate whether these findings translated to improved gut barrier function. While no significant effects were observed under unstressed conditions, GDL/NUTR blends tended to improve TEER upon LPS-induced stress. This suggests that changes in the gut metabolome by GDL and NUTR fermentation may induce beneficial effects on the gut lining, which may help support resilience under inflammatory conditions.

Finally, the limitations of this study must be acknowledged. The study cohort of six donors encompassed multiple distinct microbiota profiles and has previously been shown to be sufficient to reproduce observations from independent clinical studies (Van den Abbeele et al., 2023). Nevertheless, larger cohorts would be advantageous to further capture interindividual variability and strengthen the generalizability of the findings. Furthermore, the present work relies on preclinical colonic simulations performed *ex vivo*. Although the SIFR^®^ technology has been experimentally validated to predict microbiome shifts observed in humans (Van den Abbeele et al., 2023), it cannot fully recapitulate the complexity of the *in vivo* environment, including the dynamic interactions between the microbiota and the host. As a result, the potential health implications will require further investigation, ideally in well-controlled human intervention studies.

## 5 Conclusion

GDL and NUTR displayed compatible activities that could be integrated into blended formulations with tunable microbiome and metabolite outputs. While GDL favored rapid bifidogenic and butyrogenic responses, NUTR promoted broader microbial diversification and propionogenic activity, resulting in distinct yet complementary functional profiles. Furthermore, fermented GDL, NUTR and their blends improved gut barrier resilience under inflammatory stress conditions in a host-microbiome interaction model.

Collectively, these findings provide mechanistic insights into how structurally distinct fermentable ingredients can be combined to achieve a broader and more balanced microbiome modulation than can be obtained with individual ingredients alone. This work supports the rational design of multi-ingredient formulations that target complementary microbiome-mediated health pathways.

## Data Availability

The 16S rRNA gene profiling dataset generated during the current study has been deposited in the European Nucleotide Archive (ENA) database (Project Accession Number PRJEB123005). The metabolomics data generated in this study have been deposited in Figshare and are available at https://doi.org/10.6084/m9.figshare.33952411. Other data supporting the findings of this study are available from the corresponding author upon reasonable request.
