# Supplementary material for: Soluble prebiotic fiber and glucono-δ-lactone blends integrate compatible effects to modulate microbiota composition and metabolism ex vivo
Source: Front Microbiol. 2026 Oct 8;17:1923298. doi: 10.3389/fmicb.2026.1923298 (PMC13687269; doi:10.3389/fmicb.2026.1923298)
Supplement: Supplementary file 1 [file Data_Sheet_1.PDF]

Supplementary Material

1 Supplementary Figures

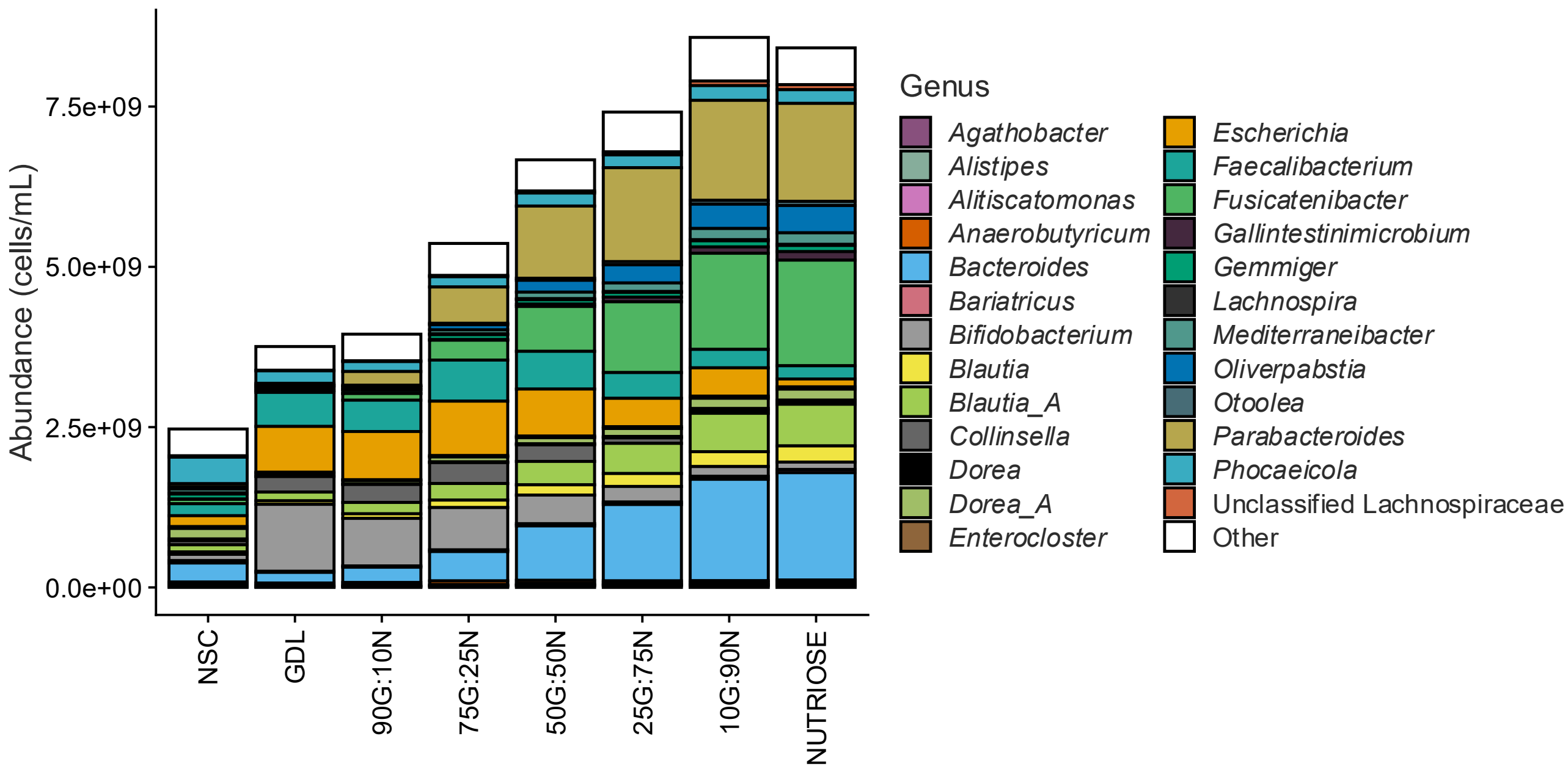

Figure S1. Glucono-δ-lactone (GDL) and NUTRIOSE® (NUTR) exerted compatible effects on genus-level microbiota composition. Average absolute abundance (cells/mL) of the 25 most abundant bacterial genera after 48h of simulated colonic fermentation by the gut microbiota of healthy adults (*n* = 6), compared to a no-substrate control (NSC).

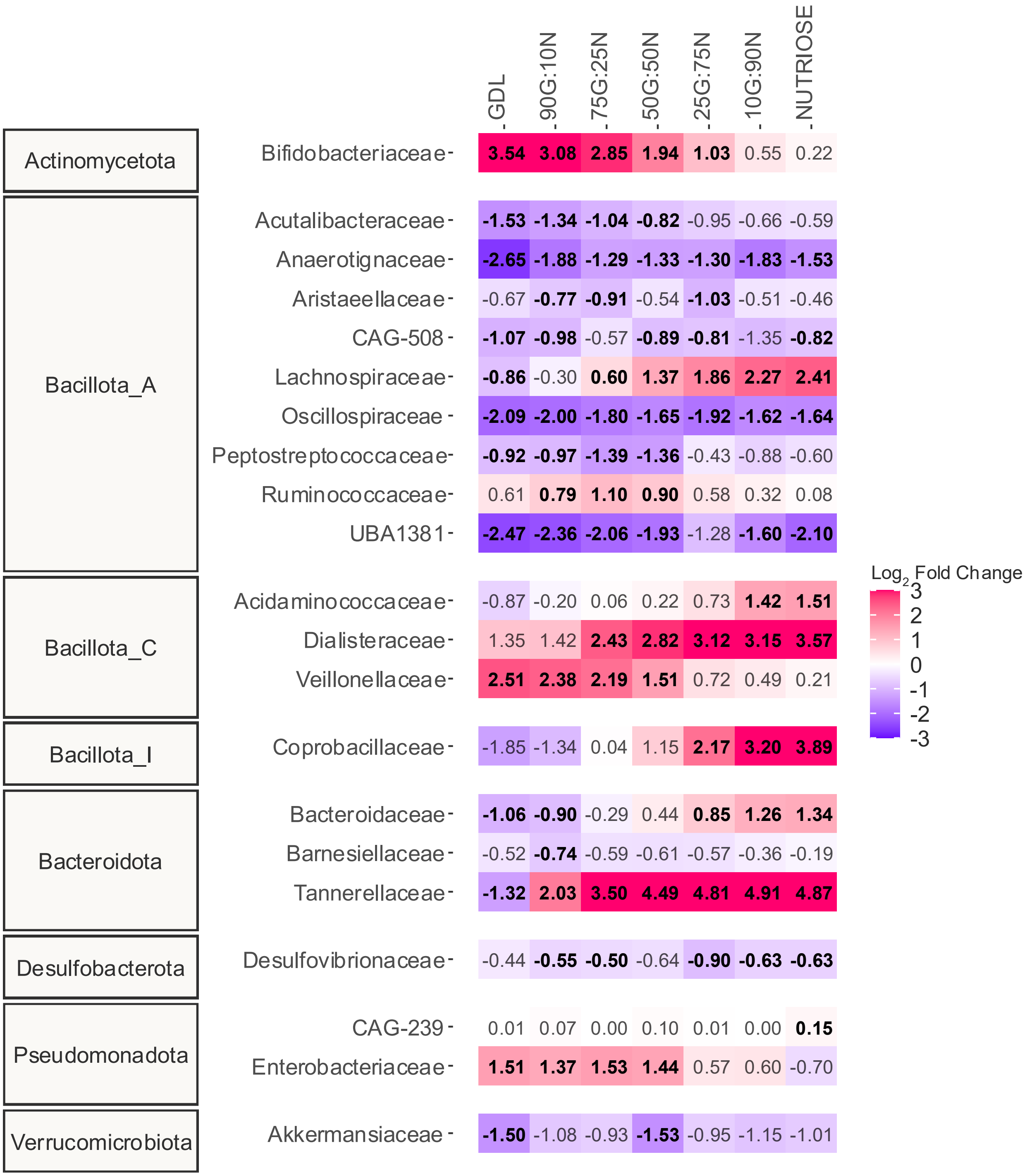

Figure S2. Glucono-δ-lactone (GDL) and NUTRIOSE® (NUTR) stimulated specific microbial families, with blends showing intermediate effects. The heatmap displays significantly affected families, expressed as $\log_2$ fold change in cell density (cells/mL) compared to the no-substrate control (NSC) after 48h of simulated colonic fermentation by the gut microbiota of healthy adults ($n = 6$). Bold numbers indicate significant treatment effects ($p_{adjusted} < 0.05$) compared to the NSC. The corresponding phyla are highlighted on the left.

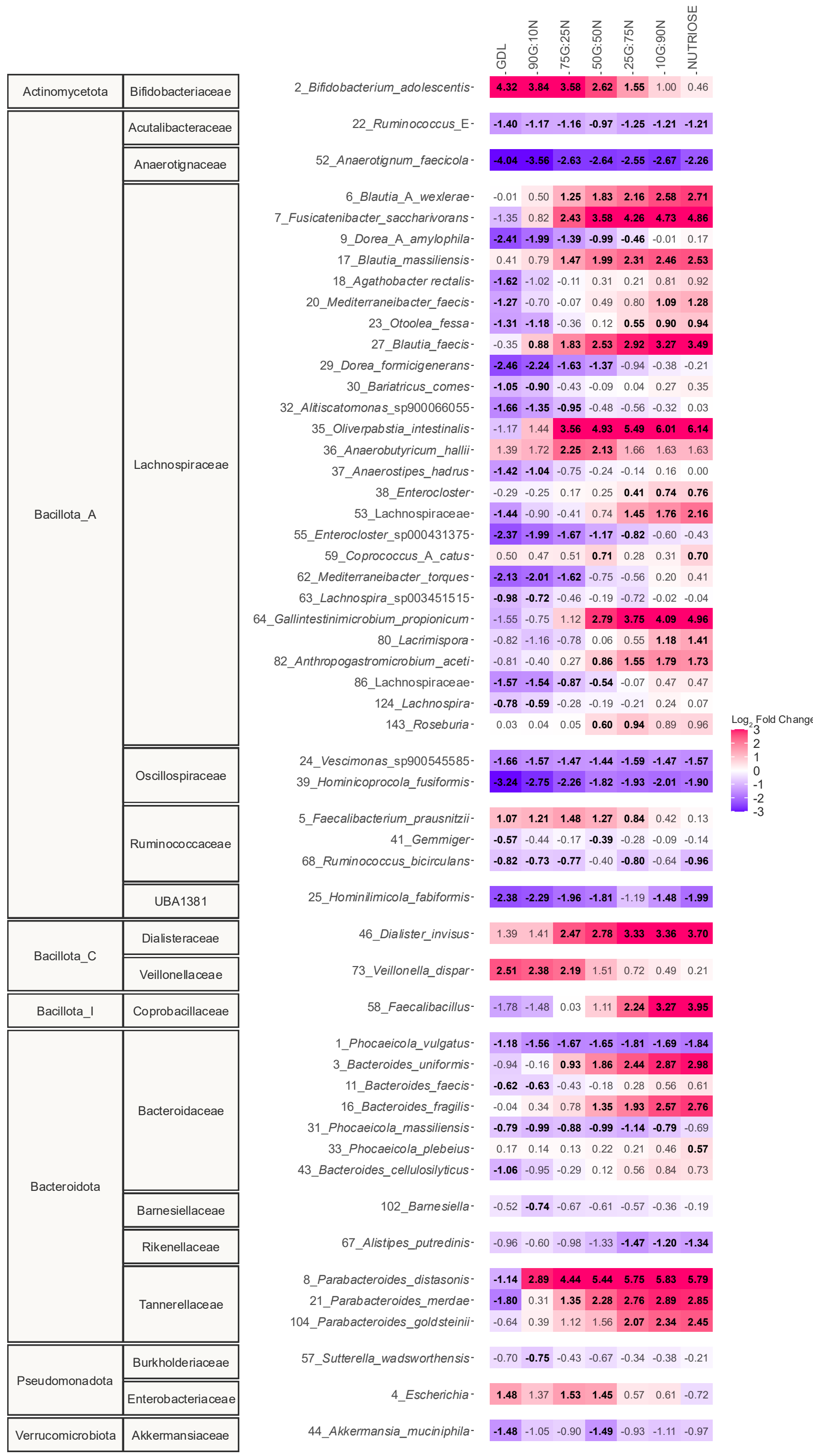

Figure S3. Glucono-δ-lactone (GDL) and NUTRIOSE® (NUTR) stimulated specific microbial OTUs, with blends showing intermediate effects. The heatmap displays significantly affected OTUs, expressed as $\log_2$ fold change in cell density (cells/mL) compared to the no-substrate control (NSC) after 48h of simulated colonic fermentation by the gut microbiota of healthy adults (n = 6). Bold numbers indicate significant treatment effects ($p_{adjusted}$ < 0.05) compared to the NSC. The corresponding phyla and families are highlighted on the left.

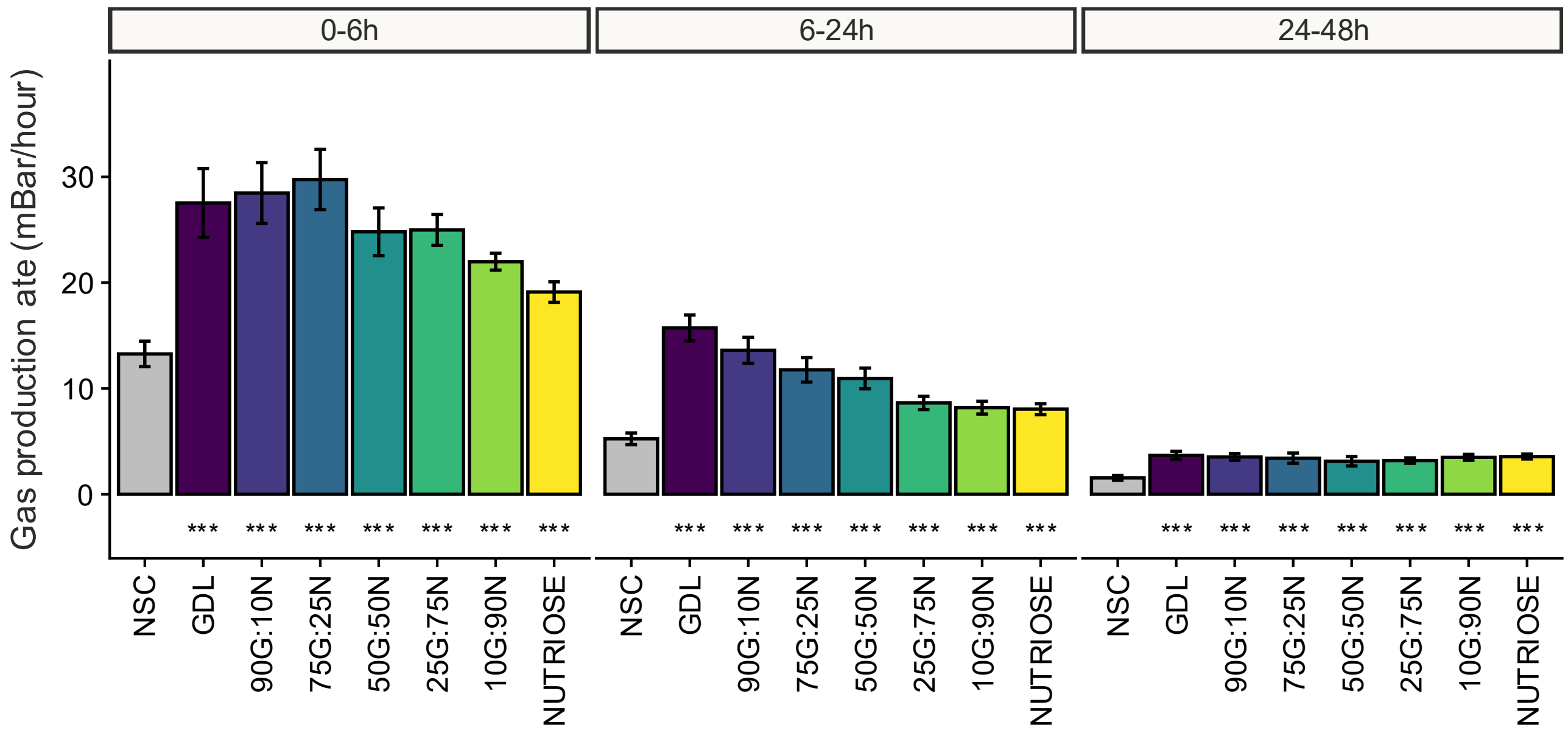

Figure S4. Glucono-δ-lactone (GDL), NUTRIOSE® (NUTR) and their blends affected gas production kinetics. Gas production rate (mBar/hour) during simulation colonic fermentation of various ratios of GDL and NUTR, or a no-substrate control (NSC), by the gut microbiota of healthy adults (n = 6). Data are presented as the differential gas pressure between consecutive timepoints (0-6h, 6-24h and 24-48h), divided by the interval duration, and averaged across the six donors. Error bars represent standard deviations. Statistical differences between NSC and individual treatments are indicated with * ($0.01 < p_{adjusted} < 0.05$), ** ($0.001 < p_{adjusted} < 0.01$) or *** ($p_{adjusted} < 0.001$).

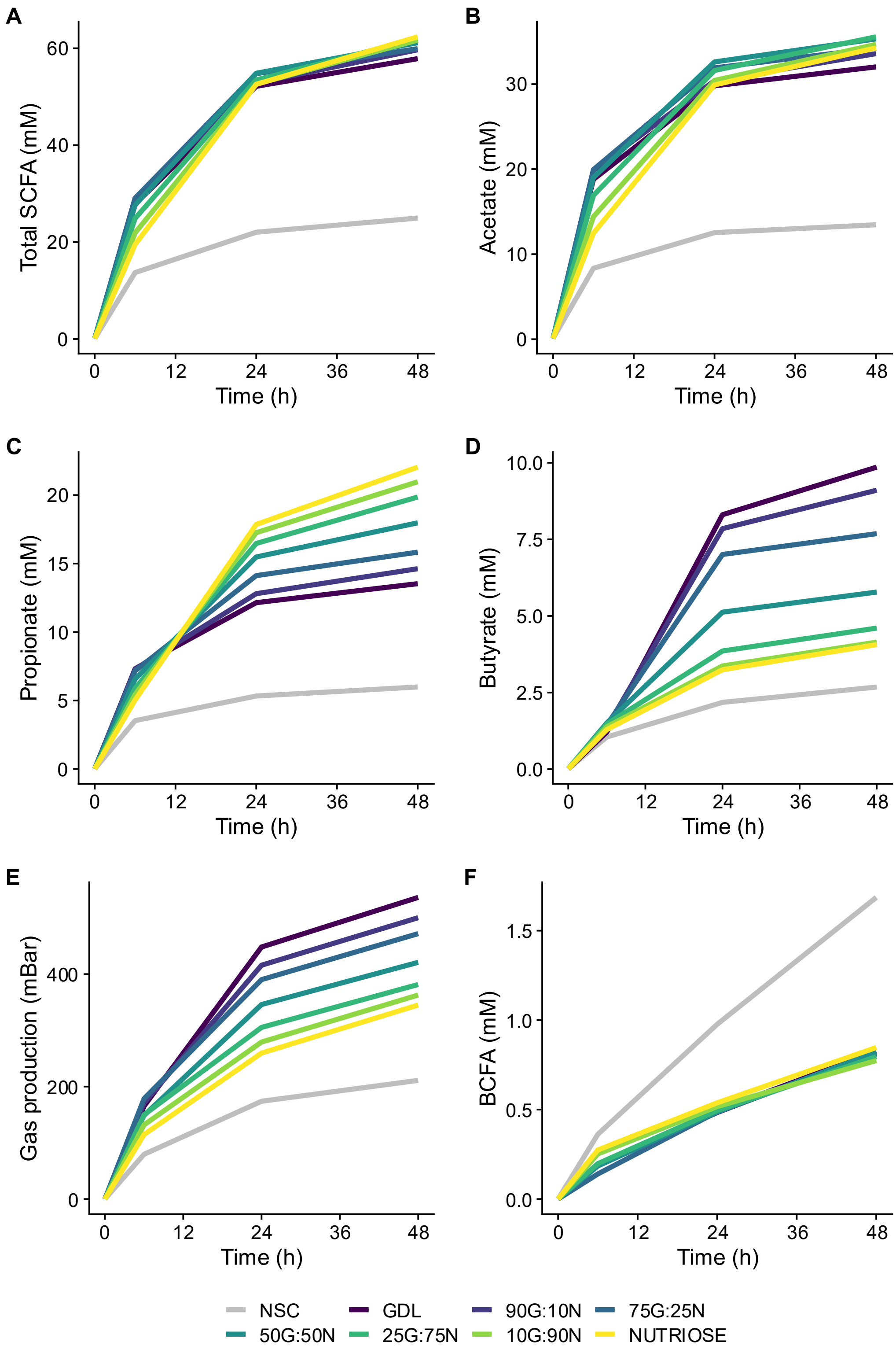

Figure S5. GDL, NUTR and blends thereof differentially affected fermentative parameters over time. Impact of glucono-δ-lactone (GDL) or NUTRIOSE® (NUTR) and their blends on (**A**) total short-chain fatty acids (SCFA), (**B**) acetate, (**C**) propionate, (**D**) butyrate, (**E**) gas and (**F**) branched-chain fatty acids (BCFA) production, at different timepoints during their simulated colonic fermentation by the gut microbiota of healthy adults (*n* = 6). Values are presented as the average among the six donors.

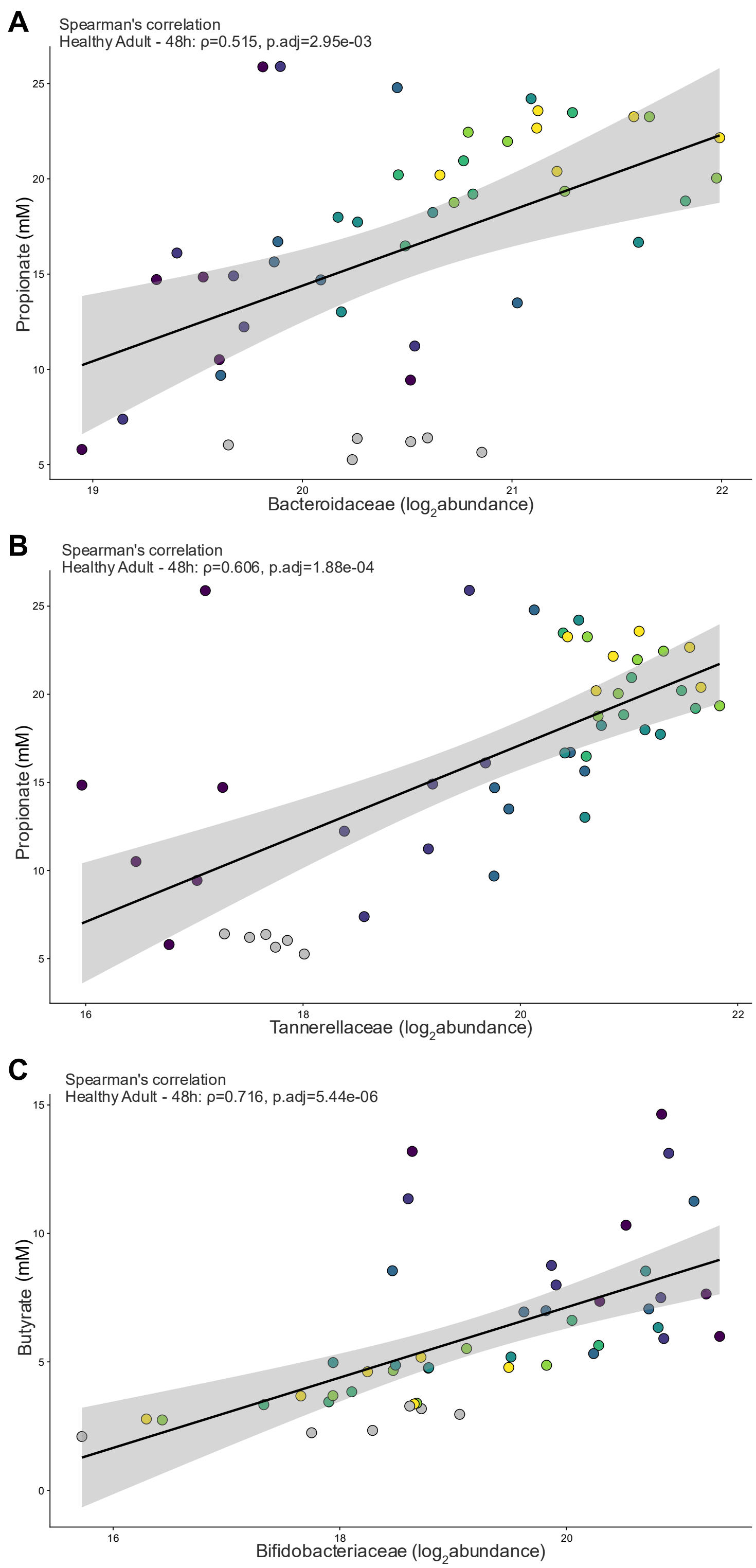

Figure S6. Propionate concentrations correlated with Bacteroidaceae and Tannerellaceae, while butyrate correlated with Bifidobacteriaceae. Correlations were assessed using Spearman's rank correlation analysis based on data obtained after 48h of simulated colonic fermentation by the gut microbiota of healthy adults (n = 6). (**A**) Propionate and Bacteroidaceae, (**B**) propionate and Tannerellaceae, and (**C**) butyrate and Bifidobacteriaceae.

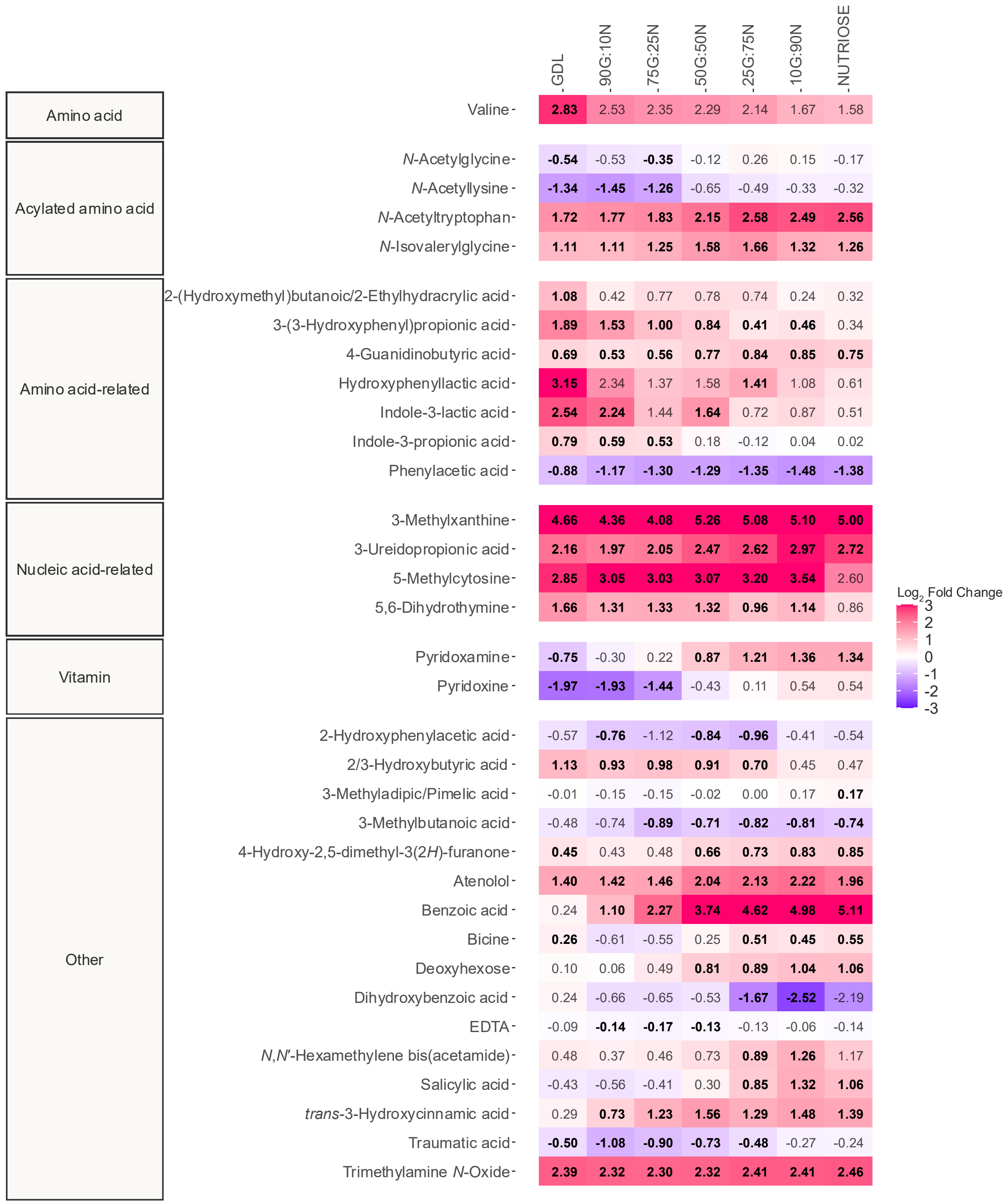

Figure S7. Glucono-δ-lactone (GDL) and NUTRIOSE® (NUTR) divergently modulated metabolite profiles, with blends showing intermediate effects. The heatmap displays significantly affected metabolites, expressed as $\log_2$ fold change in concentration (AUC) compared to the no-substrate control (NSC) after 48h of simulated colonic fermentation by the gut microbiota of healthy adults (n = 6). Bold numbers indicate significant treatment effects ($p_{adjusted} < 0.05$) compared to the NSC. Corresponding metabolite classes are highlighted on the left.

2 Supplementary Tables

Table S1. Statistical comparisons for all study endpoints. Excel file containing the results of all pairwise statistical comparisons performed throughout the study, including: (i) key fermentative parameters, (ii) gas production rate, (iii) community modulation score (CMS), (iv) abundance of bacterial taxa at phylum, family, genus and OTU level, (v) Spearman's rank correlation analysis between bacterial families and the three main SCFA, (vi) untargeted metabolomics analysis, and (vii) transepithelial electrical resistance (TEER) in a Caco-2/THP-1 co-culture under both unstressed (24h) and LPS-stressed (30h) conditions.
